# Effect of Probiotic and Synbiotic Oral Supplementation in Autoimmune Diseases: An Updated Systematic Review and Meta-Analysis of Randomized Controlled Trials

**DOI:** 10.3390/nu18071107

**Published:** 2026-03-30

**Authors:** Yuan-Yow Chiou, Tsu-Yun Chiu, Mei-Ju Chen

**Affiliations:** 1 Department of Pediatrics, Division of Pediatric Nephrology, National Cheng Kung University Hospital, College of Medicine, National Cheng Kung University, Tainan 704302, Taiwan; 2 Department of Psychology, Kaohsiung Medical University, Kaohsiung City 807378, Taiwan; 3 Department of Senior Welfare and Services, Southern Taiwan University of Science and Technology, Tainan 710301, Taiwan

**Keywords:** probiotics, autoimmune diseases, rheumatoid arthritis (RA), systemic lupus erythematosus (SLE), multiple sclerosis (MS), psoriasis

## Abstract

Background: Autoimmune diseases affect 5–10% of the global population. Probiotic supplementation has emerged as a potential adjunctive therapy in managing inflammation associated with these conditions. This systematic review and meta-analysis aimed to examine the effectiveness of oral probiotics in patients with autoimmune diseases for managing inflammation. Methods: A literature search of PubMed, EMBASE, and Cochrane CENTRAL was performed up to 18 June 2024. Eligible studies were randomized controlled trials (RCTs) examining the effects of oral supplementation of probiotics, synbiotics, or prebiotics in patients with established autoimmune diseases. The primary outcome was changes in inflammatory markers, including interleukin (IL)-6, IL-10, IL-1β, tumor necrosis factor (TNF)α, and high-sensitivity *C*-reactive protein (hs-CRP). Results: Twelve RCTs involving 703 patients were included. Significant reductions were observed in levels of IL-6 (pooled standardized mean difference [pSMD] = −0.83; 95% confidence interval [CI]: −1.30, −0.37), IL-10 (pSMD = −0.30; 95% CI: −0.61, −0.00), TNFα (pSMD = −0.41; 95% CI: −0.77, −0.06), and hs-CRP (pSMD = −0.71; 95% CI: −1.18, −0.23) in patients taking probiotic supplementation. Subgroup analysis revealed that in rheumatoid arthritis (RA) patients, the probiotics group showed greater improvements in IL-6, IL-1β, and TNFα compared to the controls. In multiple sclerosis (MS) patients, the probiotics group demonstrated greater improvements in hs-CRP. Conclusions: Oral probiotic supplementation lowers the levels of some inflammatory markers in patients with autoimmune diseases. Further studies with longer follow-up durations are needed to confirm these findings and explore the long-term benefits of probiotics in this population.

## 1. Introduction

Autoimmune diseases represent a broad spectrum of conditions in which the immune system erroneously targets the body’s own tissues, leading to chronic inflammation and organ dysfunction [1,2,3]. The global prevalence of autoimmune diseases has been estimated at around 5–10% of the population, with some evidence indicating a rising trend, especially in industrialized nations [4]. This increase is often attributed to environmental factors, lifestyle changes, genetic predispositions, and alterations in gut microbiota [5,6,7]. Over 80 different autoimmune diseases have been identified, and the most common ones are rheumatoid arthritis (RA), systemic lupus erythematosus (SLE), multiple sclerosis (MS), psoriasis, inflammatory bowel disease (IBD), and type 1 diabetes (T1D) [8]. Autoimmune diseases impose a substantial burden on healthcare systems worldwide. The cost associated with autoimmune diseases includes direct expenses such as medications, hospitalizations, and physician visits, and indirect costs like loss of productivity and reduced work capacity. In the United States, the annual economic burden of autoimmune diseases is estimated to exceed $100 billion, surpassing the healthcare costs associated with cancer [9]. This significant economic impact underscores the need for effective, accessible, and sustainable treatments.

The prognosis of autoimmune diseases varies considerably depending on the specific condition and its severity. For many patients, autoimmune diseases are lifelong and require ongoing management to control symptoms and prevent disease progression [10]. Some autoimmune diseases such as RA and SLE are associated with significant morbidity, reduced quality of life, and increased mortality, particularly when not adequately managed [11,12,13,14]. Furthermore, autoimmune diseases are often characterized by periods of remission and relapse, posing challenges in achieving long-term disease control.

Research into the gut–immune axis has shed light on the pivotal role of the gut microbiota in immune system regulation and the potential development of autoimmune diseases [15,16]. An imbalance in gut microbiota, known as dysbiosis, has been associated with the onset and progression of several autoimmune conditions. Notably, studies have shown that probiotics have a positive impact on health and the outcomes of many diseases [17,18,19]. In addition, the use of probiotics as adjunctive therapy in autoimmune diseases has been explored. In patients with RA, probiotics have been found to potentially to modulate inflammatory pathways, and previous meta-analyses have shown that probiotics improve outcomes in patients with RA [20,21,22,23,24,25]. However, in conditions like SLE, MS, and psoriasis, the effects of probiotics are less well recognized, and the evidence for a positive effect is conflicting. These findings suggest that while probiotics hold promise as complementary therapies for autoimmune diseases, there remains a need for more comprehensive analyses to establish their efficacy.

Therefore, the purpose of this study is to perform a systematic review and meta-analysis to synthesize current evidence and clarify the effectiveness of probiotics in treating autoimmune diseases.

## 2. Methods

### 2.1. Search Strategy

We conducted this systematic review and meta-analysis according to PRISMA guidance [26]. PubMed, EMBASE, and Cochrane CENTRAL were searched from inception to 18 June 2024, using intervention-related terms (“probiotics,” “synbiotics,” and “prebiotics”) and disease-related terms (“autoimmune disease,” “rheumatoid arthritis,” “systemic lupus erythematosus,” “multiple sclerosis,” and “psoriasis”), with Boolean logic and MeSH terms where applicable. The detailed search string was:

(Synbiotics OR probiotics OR prebiotics OR Lactobacillus)

AND

(‘autoimmune disease’ OR ‘rheumatoid arthritis’ OR ‘systemic lupus erythematosus’ OR ‘multiple sclerosis’ OR ‘psoriasis’ OR ‘autoimmune thyroiditis’ OR ‘Sjogren’s syndrome’)

AND

(‘hs-CRP’ OR ‘IL-10’ OR ‘IL-6’ OR ‘IL-1β’ OR ‘TNF-a’ OR ‘ESR’ OR ‘GSH’ OR ‘MDA’ OR ‘TAC’ OR ‘HOMA-IR’ OR ‘HOMA-b’ OR ‘disease activity’ OR pain)

In addition, the reference lists of included studies were hand-searched to identify other potentially relevant studies.

### 2.2. Selection Criteria

Eligibility was defined through the PICOS framework (participants, interventions, comparators, outcomes, and study design). We included studies enrolling patients with autoimmune diseases, including RA, SLE, MS, psoriasis, and Sjogren syndrome. Eligible interventions were oral supplementation with probiotics, synbiotics, or prebiotics. Comparators comprised placebo, no active treatment, or dietary interventions that did not include probiotics, synbiotics, or prebiotics. Outcomes of interest were quantitative changes in at least one inflammatory or oxidative stress-related biomarker, such as interleukin (IL) concentrations or other oxidative stress indices. We restricted inclusion to published randomized controlled trials with either open-label or blinded designs.

We excluded non-original or non-eligible publication types (reviews, letters, commentaries, editorials, case reports, conference proceedings/abstracts, and personal communications), as well as non-randomized studies, non-human studies, and studies lacking quantitative data for the prespecified outcomes of interest. Study eligibility was assessed independently by two reviewers based on the predefined search and selection criteria; any discrepancies or uncertainties were resolved through discussion, with adjudication by a third reviewer when necessary.

This systematic review and meta-analysis is registered in the International Prospective Register of Systematic Reviews (PROSPERO) under the registration number CRD420251116568.

### 2.3. Main Outcome Measures and Data Extraction

The primary outcomes were circulating levels of IL-6, IL-10, IL-1β, tumor necrosis factor (TNF)α, and high sensitivity *C*-reactive protein (hs-CRP). Secondary outcomes included malondialdehyde (MDA) and total antioxidant capacity (TAC). Treatment effects were quantified using the standardized mean difference (SMD) based on changes from baseline. When the standard deviation (SD) was not reported and only the mean and range were available, the SD was derived using an established approximation method:

$$SD\approx \frac{Range}{4}.$$

Changes from baseline were calculated as the post-intervention value minus the baseline value within each study arm, and the meta-analysis pooled the between-group difference in these change scores using SMD. A negative SMD indicates a greater decrease (i.e., larger reduction from baseline) in the intervention group than in the control group, whereas a positive SMD indicates a greater increase (or a smaller reduction) in the intervention group relative to the control group. For biomarkers where lower values reflect improvement (e.g., IL-6, IL-10, IL-1β, TNFα, hs-CRP, and MDA), a negative SMD was interpreted as a favorable effect; for biomarkers where higher values reflect improvement (e.g., TAC), a positive SMD was interpreted as favorable.

From each eligible study, we extracted study-level characteristics including first author, publication year, country/setting, target population, participant age, proportion of male participants, total sample size, and the numbers allocated to the intervention and control arms. We also recorded trial duration, detailed intervention and comparator protocols (including dosage and supplementation period), and all reported outcomes relevant to this review. Extracted data were compiled and managed in Microsoft Excel.

### 2.4. Risk of Bias Assessment

The quality of included studies was assessed using the Cochrane Collaboration tool [27]. Risk of bias was assessed across seven domains: random sequence generation and allocation concealment (selection bias), blinding of participants and personnel (performance bias), blinding of outcome assessment (detection bias), incomplete outcome data (attrition bias), selective outcome reporting (reporting bias), and other potential sources of bias. Quality assessment was performed by 2 independent reviewers, and a third reviewer was consulted if any uncertainties occurred.

### 2.5. Statistical Analysis

Effect estimates were synthesized as SMD with corresponding 95% confidence interval (CI). Between-study heterogeneity was assessed using Cochran’s Q test and the *I*^2^ statistic, which quantifies the proportion of total variability attributable to heterogeneity rather than sampling error. We interpreted *I*^2^ values as follows: *I*^2^ ≤ 25% (low heterogeneity), 25% < *I*^2^ < 50% (moderate heterogeneity), 50% < *I*^2^ < 75% (substantial heterogeneity), and *I*^2^ ≥ 75% (high heterogeneity). To account for between-study variability, we applied a random-effects model when *I*^2^ exceeded 25%; otherwise, a fixed-effects model was used when heterogeneity was low (*I*^2^ ≤ 25%). All statistical tests were two-sided, and *p* ≤ 0.05 was considered statistically significant. Potential publication bias was evaluated by visual inspection of funnel plots and formally tested using Egger’s regression test. All analyses were performed in RStudio (version 4.3.2) using the “meta,” “dmetar,” and “metafor” packages.

## 3. Results

### 3.1. Search Results

**Figure 1:**
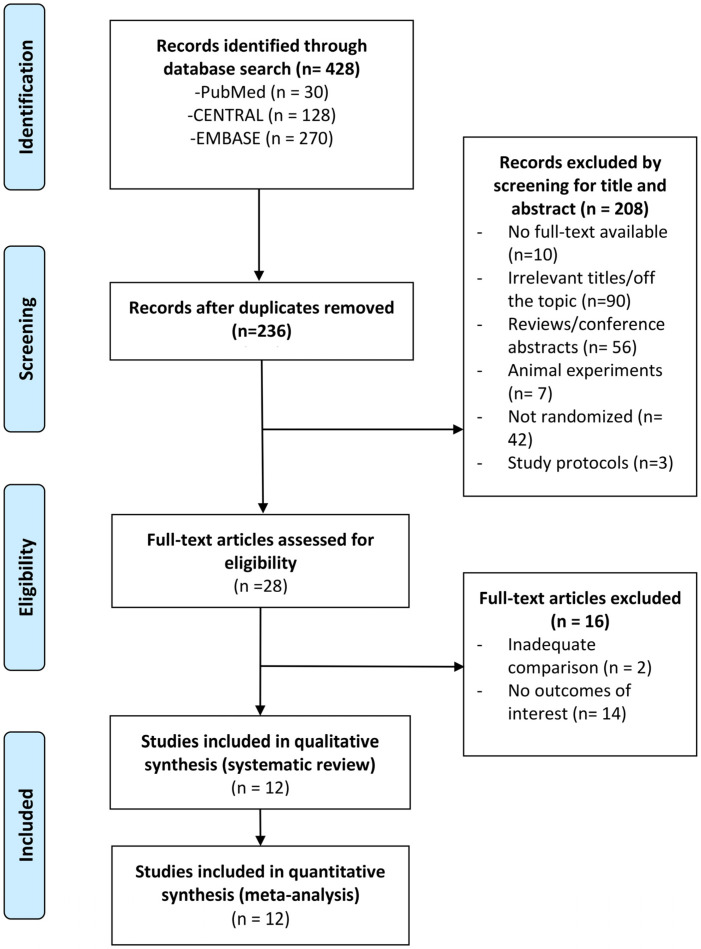
Flow diagram.

A flow diagram of the study selection process is shown in Figure 1. The database searches identified 428 entries. After removing duplicates and irrelevant studies after screening the titles and abstract, the remaining 28 studies underwent full-text review based on the inclusion and exclusion criteria. In the end, 12 RCTs were included in the systematic review and meta-analysis, encompassing a total of 703 patients [28,29,30,31,32,33,34,35,36,37,38,39] (Figure 1).

### 3.2. Study Characteristics

The characteristics of the 12 studies are summarized in Table 1. The numbers of patients ranged from 29 to 96. Four studies focused on patients with RA, three on MS, one on spondyloarthritis, one on T1D, one on SLE, and one on psoriasis. Additionally, one study included patients with ulcerative colitis (UC), chronic fatigue syndrome (CFS), and psoriasis. In addition, only one study [38] evaluated synbiotics and the other studies focused on probiotics. No eligible RCT evaluating an isolated prebiotic intervention was identified. Accordingly, the pooled estimates in the present meta-analysis are driven predominantly by probiotic data, with only limited evidence for synbiotics and no direct clinical evidence for prebiotics. The mean age of the patients ranged from 12.7 to 75.0 years, the proportion of male patients ranged from 0% to 63.0%, and the last timepoints of evaluation varied from 6 weeks to 52 weeks (Table 1).

**Table 1:** Characteristics of the included studies.

First Author (Publication Year)	Country	N	Diseases	Mean Age, Years	Male, %	Trial Duration	Intervention: Type, Dose, Frequency	Control: Type, Dose, Frequency	Probiotic Contents	Inflammatory Marker Outcome Evaluated
Jenks (2010) [33]	New Zealand	63	Spondyloarthritis	43.3	63.0	12 weeks	Probiotic powder, 0.8 g, twice daily	Placebo powder, 0.8 g, twice daily	*Streptococcus salivarius* K12 *Bifidobacterium lactis* LAFTI B94 *Lactobacillus acidophilus* LAFTI L10	CRP, Fecal calprotectin
Pineda (2011) [36]	Canada	29	RA	61.5	6.9	3 months	Probiotic capsule, twice daily for 90 days	Placebo capsule, twice daily	*Lactobacillus rhamnosus* GR-1 *Lactobacillus reuteri* RC-14	ESR, CRP, IL-1a, IL-1b, IL-6, IL-8, TNF-a, IL-12p70, IL-15, IL-10, GM-CSF, G-CSF, IL-17, sCD40 ligand, MIP-1a, MIP-1b, MCP-1
Groeger (2013) [31]	Ireland	96	UC, CFS, psoriasis	Range: 18–75	NA	8 weeks for CFS, psoriasis, and healthy controls, and 6 weeks for UC	Probiotic sachet, once daily	Placebo sachet, once daily	*Bifidobacterium infantis* 35624	CRP, IL-6, TNF-α
Alipour (2014) [28]	Iran	46	RA	42.8	0.0	8 weeks	Probiotic hard gelatin capsule, once daily	Placebo hard gelatin capsule, daily	*Lactobacillus casei* 01	hs-CRP, IL-1β, IL-6, IL-10, IL-12, TNF-α
Zamani (2016) [39]	Iran	60	RA	51.4	15.0	8 weeks	Probiotic capsule, once daily	Placebo capsule, once daily	*Lactobacillus acidophilus* *Lactobacillus casei* *Bifidobacterium bifidum*	hs-CRP, insulin, HOMA-B
Kouchaki (2017) [34]	Iran	60	MS	34.1	16.7	12 weeks	Probiotic capsule, once daily	Placebo capsule, once daily	*Lactobacillus acidophilus* *Lactobacillus casei* *Bifidobacterium bifidum* *Lactobacillus fermentum*	hs-CRP, NO, TAC, GSH, MDA
Cannarella (2021) [30]	Brazil	42	RA	57.0	11.9	60 days	Probiotic sachet, once	Placebo sachet, once daily	*Lactobacillus acidophilus* LA-14 *Lactobacillus casei* LC-11 *Lactococcus lactis* LL-23 *Bifidobacterium lactis* BL-04 *Bifidobacterium bifidum* BB-06	TNF-α, IL-6, IL-10, adiponectin, hsCRP, ESR, ferritin, WBC count
Groele (2021) [32]	Poland	96	T1D	12.7	57.3	12 months	Probiotic capsule, once daily for 6 months	Placebo capsule, once daily	*Lactobacillus rhamnosus* GG *Bifidobacterium lactis* Bb12	IL-1β, IL-2, IL-10, TNF-α, IFN-γ
Moludi (2021) [35]	Iran	50	Psoriasis	42.9	36.0	8 weeks	Probiotic capsule, twice daily	Placebo capsule, twice daily	*Lactobacillus acidophilus* *Bifidobacterium bifidum* *Bifidobacterium lactis* *Bifidobacterium langum*	MDA, hs-CRP, IL-6, TAC
Rahimlou (2022) [37]	Iran	65	MS	41.0	27.7	6 months	Two probiotic capsules, daily	Two placebo capsules, daily	*Bacillus subtilis* PXN 21 *Bifidobacterium bifidum* PXN 23 *Bifidobacterium breve* PXN 25 *Bifidobacterium infantis* PXN 27 *Bifidobacterium longum* PXN 30 *Lactobacillus acidophilus* PXN 35 *Lactobacillus delbrueckii* ssp. *Bulgaricus* PXN 39 *Lactobacillus casei* PXN 37 *Lactobacillus plantarum* PXN 47 *Lactobacillus rhamnosus* PXN 54 *Lactobacillus helveticus* PXN 45 *Lactobacillus salivarius* PXN 57 *Lactococcus lactis* ssp. *Lactis* PXN 63 *Streptococcus thermophilus* PXN 66	IL-6, BDNF, NGF
Widhani (2022) [38]	Indonesia	46	SLE	32.5	0.0	60 days	Synbiotic capsule, once daily (Prebiotic contents: Fructo-oligosaccharides, 80 mg per capsule)	Placebo capsule, once daily	*Lactobacillus helveticus* R0052 *Bifidobacterium infantis* R0033 *Bifidobacterium bifidum* R0071	hs-CRP, IL-6, IL-17
Asghari (2023) [29]	Iran	50	MS	34.4	30.0	16 weeks	Probiotic yeast capsule (BioDigest^®^), once daily	Placebo capsule containing maltodextrin, daily	*Saccharomyces boulardii*	hs-CRP, TAC, MDA

Abbreviations: N, total number of patients; RA, rheumatoid arthritis; MS, multiple sclerosis; T1D, type 1 diabetes; SLE, systemic lupus erythematosus; UC, ulcerative colitis, CFS, chronic fatigue syndrome; CRP, *C*-reactive protein; ESR, erythrocyte sedimentation rate; IL, interleukin; TNF, tumor necrosis factor; GM-CSF, granulocyte-macrophage colony-stimulating factor; G-CSF, granulocyte colony-stimulating factor; sCD40 ligand, soluble CD40 ligand; MIP-1a, macrophage inflammatory protein-1 alpha; MIP-1b, macrophage inflammatory protein-1 beta; MCP-1, monocyte chemoattractant protein-1; hs-CRP, high-sensitivity *C*-reactive protein; NO, nitric oxide; TAC, total antioxidant capacity; GSH, glutathione; MDA, malondialdehyde; IFN-γ, interferon-gamma; BDNF, brain-derived neurotrophic factor; NGF, nerve growth factor.

### 3.3. Primary Outcomes

#### 3.3.1. IL-6

**Figure 2:**
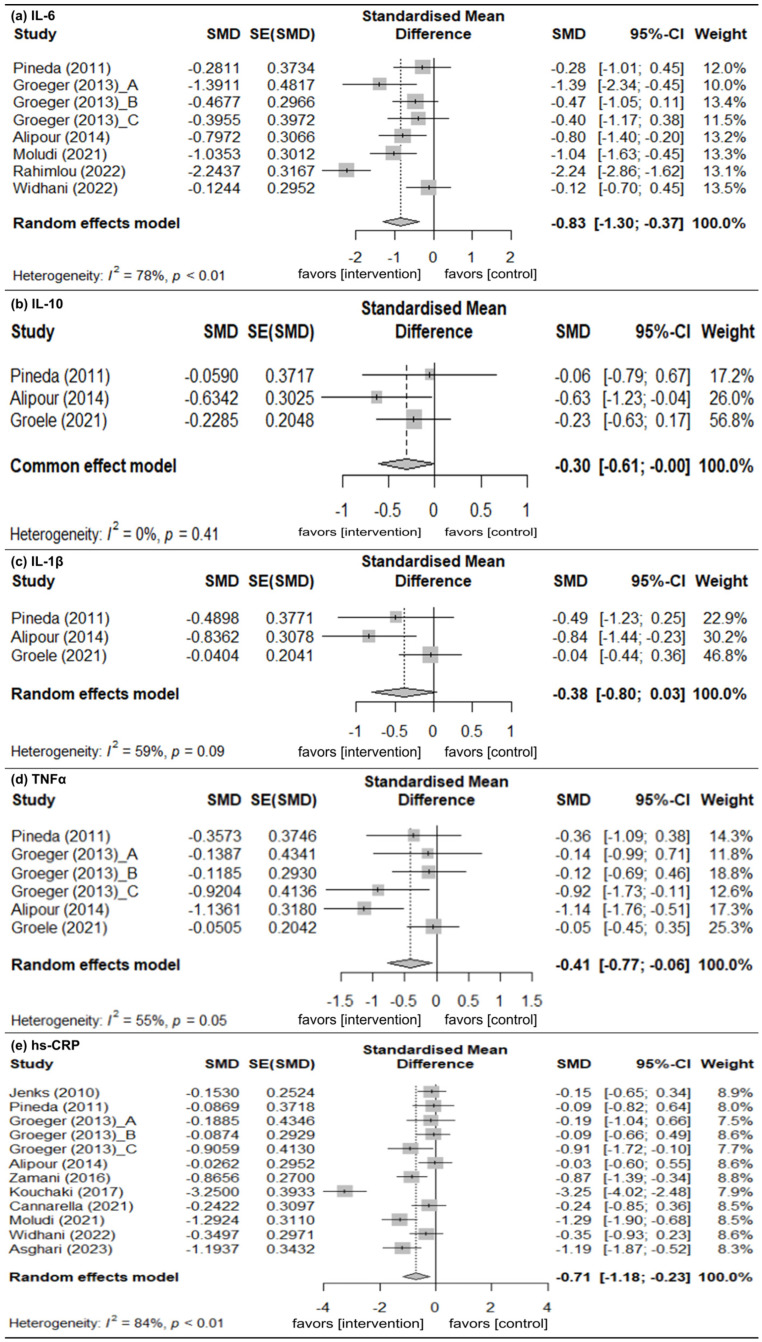
Change in inflammatory marker levels from baseline. (**a**) IL-6. (**b**) IL-10. (**c**) IL-1β. (**d**) TNFα. (**e**) hs-CRP. Gray squares represent the point estimates of individual studies (with size proportional to study weight), horizontal lines indicate the 95% confidence intervals, and the diamond represents the pooled estimate. The included references are as follows: [28,29,30,31,32,33,34,35,36,37,38,39].

Six studies provided data of the change in IL-6 levels [28,31,35,36,37,38]. A random-effects model of analysis was used (*I*^2^ = 78%). There were significant mean differences in the change in IL-6 level (pooled SMD [pSMD] = −0.83; 95% CI: −1.30, −0.37) indicating that the intervention group had significantly greater improvement than the control group (Figure 2a).

#### 3.3.2. IL-10

Three studies provided data of change in IL-10 levels [28,32,36]. A fixed-effects model of analysis was used (*I*^2^ = 0%). There were significant mean differences in the change in IL-10 level (pSMD = −0.30; 95% CI: −0.61, −0.00), indicating that the intervention group had significantly greater improvement than the control group (Figure 2b).

#### 3.3.3. IL-1β

Three studies provided data of the change in IL-1β levels [28,32,36]. A random-effects model of analysis was used (*I*^2^ = 59%). There were no significant mean differences in the change in IL-1β level (pSMD = −0.38; 95% CI: −0.80, 0.03), indicating that there was no difference in change between the intervention and control groups (Figure 2c).

#### 3.3.4. TNFα

Four studies provided data of the change in TNFα levels [28,31,32,36]. A random-effects model of analysis was used (*I*^2^ = 55%). There were significant mean differences in the change in TNFα levels (pSMD = −0.41; 95% CI: −0.77, −0.06), indicating that the intervention group had significantly greater improvement than the control group (Figure 2d).

#### 3.3.5. hs-CRP

Ten studies reported data of change in hs-CRP levels [28,29,30,31,33,34,35,36,38,39]. A random-effects model of analysis was used (*I*^2^ = 84%). There were significant mean differences in the change in hs-CRP levels (pSMD = −0.71; 95% CI: −1.18, −0.23), indicating that the intervention group had significantly greater improvement than the control group (Figure 2e).

### 3.4. Secondary Outcomes

#### 3.4.1. MDA

**Figure 3:**
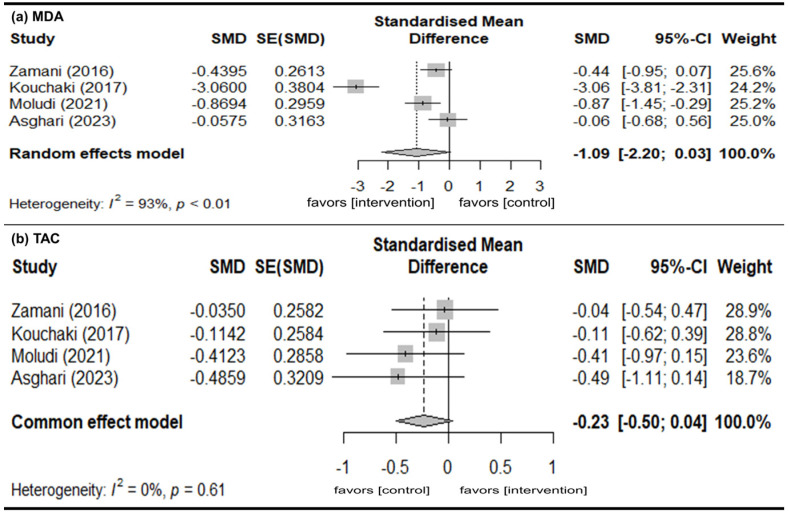
Change in inflammatory marker levels from baseline. (**a**) MDA. (**b**) TAC. Gray squares represent the point estimates of individual studies (with size proportional to study weight), horizontal lines indicate the 95% confidence intervals, and the diamond represents the pooled estimate. The included references are as follows: [29,34,35,39].

Four studies provided data of the change in MDA levels [29,34,35,39]. A random-effects model of analysis was used (*I*^2^ = 93%). There were no significant mean differences in the change in MDA levels (pSMD = −1.09; 95% CI: −2.20, 0.03), indicating no difference between the intervention and control groups (Figure 3a).

#### 3.4.2. TAC

Four studies reported data on the change in TAC levels [29,34,35,39]. A fixed-effects model of analysis was used (*I*^2^ = 0%). There were no significant mean differences in the change in TAC levels (pSMD = −0.23; 95% CI: −0.50, 0.04), indicating that there was no difference between the intervention and control groups (Figure 3b).

### 3.5. Publication Bias Analysis

**Figure 4:**
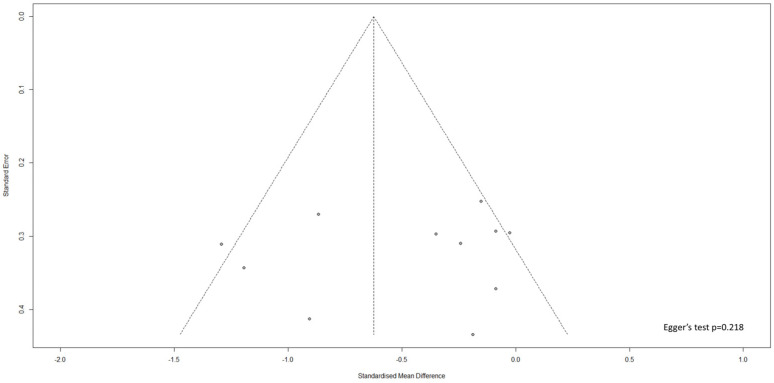
Funnel plot assessing publication bias for studies reporting changes in hs-CRP from baseline.

The funnel plot for evaluation of publication bias for the studies examining change in hs-CRP levels is shown in Figure 4. Studies examining the change in hs-CRP levels did not show evidence of publication bias according to Egger’s test (*p* = 0.218; Figure 4).

### 3.6. Subgroup Analysis of Patients with RA

**Figure 5:**
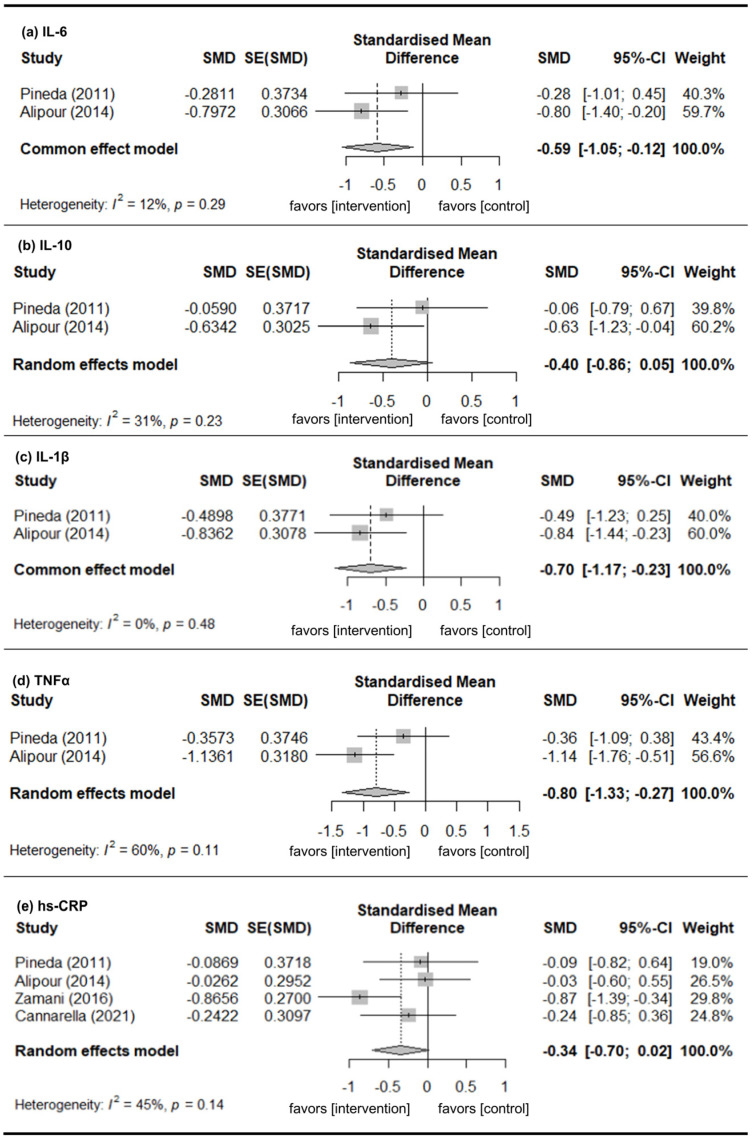
Change in inflammatory marker levels from baseline in patients with RA. (**a**) IL-6. (**b**) IL-10. (**c**) IL-1β. (**d**) TNFα. (**e**) hs-CRP. Gray squares represent the point estimates of individual studies (with size proportional to study weight), horizontal lines indicate the 95% confidence intervals, and the diamond represents the pooled estimate. The included references are as follows: [28,30,36,39].

In the studies focusing on RA, there were significant mean differences in changes in IL-6, IL-1β, and TNFα level, indicating that the intervention group improved more than the control group (Figure 5a,c,d). However, no significant mean differences were observed for the change in IL-10 and hs-CRP levels from baseline (Figure 5b,e).

### 3.7. Subgroup Analysis for Patients with MS

**Figure 6:**
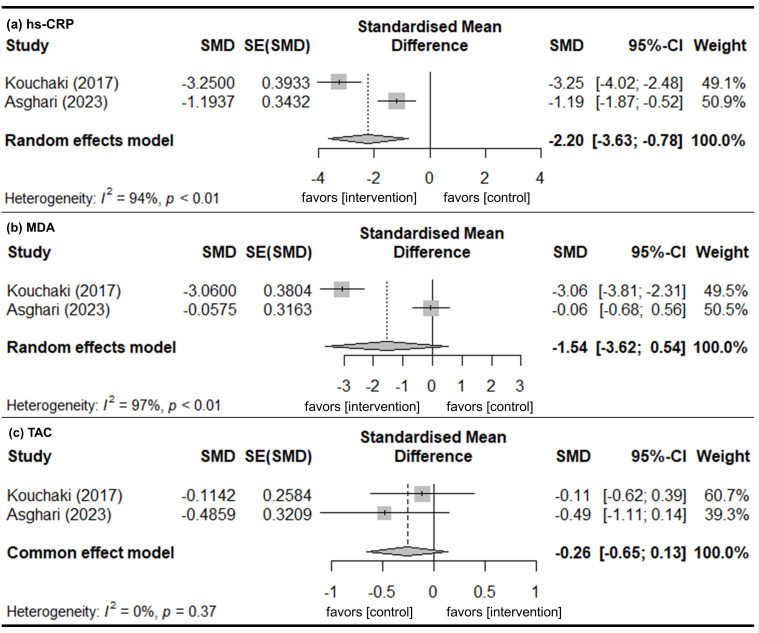
Change in inflammatory marker levels from baseline in patients with MS. (**a**) hs-CRP. (**b**) MDA. (**c**) TAC. Gray squares represent the point estimates of individual studies (with size proportional to study weight), horizontal lines indicate the 95% confidence intervals, and the diamond represents the pooled estimate. The included references are as follows: [29,34].

In studies of patients with MS, there were significant mean differences of change in hs-CRP levels, indicating that the intervention group improved more than the control group (Figure 6a). However, no significant mean differences were observed for changes in MDA and TAC from baseline (Figure 6b,c).

### 3.8. Risk of Bias Assessment

The results of the quality assessment for the included studies are presented in Supplementary Figure S1. Overall, most domains were rated as low risk of bias; unclear risk was mainly related to the blinding of outcome assessment in five trials (5/12 studies, 41.7%); no domain was rated as high risk of bias (Supplementary Figure S1A,B).

## 4. Discussion

This updated systematic review and meta-analysis evaluated the effect of oral probiotic supplementation in patients with autoimmune diseases. The analysis included 12 randomized controlled trials (RCTs) with a total of 703 participants. The primary outcomes assessed were levels of inflammatory markers IL-6, IL-10, IL-1β, TNFα, and hs-CRP. Significant reductions were observed in IL-6, IL-10, TNFα, and hs-CRP levels in patients taking probiotics compared to control groups. However, no significant changes were found for IL-1β. Secondary outcomes showed no significant differences in MDA and TAC. Subgroup analysis showed that for patients with RA, probiotics effectively reduced IL-6, IL-1β, and TNFα levels, while no significant changes were noted for IL-10 and hs-CRP. In addition, greater improvement in hs-CRP was observed in the MS patient subgroup with probiotic supplementation. Overall, these findings enhance the reliability of previous meta-analyses that suggest that oral probiotic supplementation may be a promising adjunctive therapy for managing inflammation in autoimmune diseases, especially RA.

The gut microbiota is composed of millions of different microbes, and in recent decades research has shown its importance in health and disease [40,41]. A “normal” gut microbiota composition is associated with generally good health and freedom from disease; however, microbiota dysbiosis, an abnormal composition of microbes in the gut, has been linked to many human diseases such as depression, hypertension, cardiovascular disease, obesity, diabetes, inflammatory bowel disease, and cancers [40,41]. Probiotics are active microbial species that help to reverse gut microbiota dysbiosis and return the microbiota to a healthy state [42,43,44,45,46]. Studies have shown that improvement of the gut microbiota with probiotics restores the function of the immune system and alleviates the symptoms of many diseases [44,45,46,47]. A large amount of research is being devoted to the gut microbiota, autoimmune diseases, and probiotics [47,48,49,50]. It is believed that microbiota dysbiosis leads to loss of immune tolerance, overactivation of T cells, and production of various pro-inflammatory cytokines [48,49,50,51]. These changes can lead to the development of different diseases, including autoimmune diseases [48,49,50]. Notably, overgrowth of different microbes in the gut microbiota has been associated with the development of specific autoimmune diseases [48].

This updated systematic review and meta-analysis underscores the growing interest in the role of probiotics as adjunctive therapy for managing inflammation in autoimmune diseases. It builds on previous meta-analyses by incorporating more recent trials, specifically RCTs. Prior meta-analyses have evaluated the effect of probiotics on autoimmune diseases, and our results are consistent with those of prior studies showing that probiotics improve the levels of inflammatory markers. In addition, a subgroup analysis of patients with RA showed that probiotics resulted in significant mean differences in changes in IL-6, IL-1β, and TNFα levels, and in patients with MS, probiotics resulted in significant changes in hs-CRP levels.

RA is a debilitating chronic inflammatory autoimmune disease that primarily affects the joints. It causes pain, swelling, and stiffness of the joints, and can eventually lead to severe joint damage, disability, and other health problems such as heart disease, lung disease, and skin problems. A number of other reviews and meta-analyses have been published examining the effect of probiotics on RA. However, it should be noted that in all of these reviews and meta-analyses, the authors indicated large heterogeneity between studies, and it was difficult to draw definitive conclusions. In a systematic review published in 2020, Lowe et al. [22] examined the effect of probiotics in patients with inflammatory arthritis. Their results showed that probiotics had a statistically significant benefit on quality of life (SMD = −0.37), a small but significant reduction in pain (MD = −8.97), and a significant reduction in CRP level (MD = −2.33). Notably, the greatest effect on RA was achieved with combined *Bifidobacteriales* and *Lactobacillales* formulations.

A systematic review and meta-analysis published in 2021 found that probiotic and synbiotic supplementation reduced the levels of IL-6, TNFα, hs-CRP, and MDA, as well as HOMA-IR in patients with RA [21]. A similar study published in 2022, which included eight studies with a total of 344 patients with RA, found that probiotic supplementation only reduced the CRP level [24]. Wu et al. [25] performed a systematic review and meta-analysis of the effect of probiotics in patients with RA. A total of 12 RCTs were included in the analysis, and the results indicated that probiotics improved the disease activity score (DAS) and reduced the levels of inflammatory mediators such as CRP and IL-1B. There was no effect on VAS pain score or ESR.

In RA, a self-amplifying inflammatory network dominated by TNF-α, IL-1β, and IL-6 sustains synovial macrophage and fibroblast-like synoviocyte activation, promotes leukocyte recruitment, and propagates local joint damage and systemic inflammation [52,53]. Beyond the joint, accumulating evidence supports a “gut–joint axis,” in which intestinal dysbiosis and impaired epithelial barrier integrity increase the translocation of microbial products (for example, lipopolysaccharide), thereby enhancing innate immune signaling and skewing adaptive immunity toward T helper 17 cell (Th17)-dominant responses [54,55]. Probiotics may mitigate these processes by remodeling the microbial ecosystem, improving barrier function, and increasing immunoregulatory metabolites (such as butyrate) that promote regulatory T cells (Treg) and suppress autoimmune arthritis pathways, which would be expected to preferentially downshift proximal pro-inflammatory cytokines (IL-6, IL-1β, and TNF-α), consistent with prior randomized trials and evidence syntheses in rheumatoid arthritis [24,56]. In contrast, IL-10 may remain unchanged because its induction is strain-, tissue-, and timing-dependent and circulating levels may not capture local mucosal or synovial immunoregulation; similarly, hs-CRP is a hepatic acute-phase reactant whose expression depends on cytokine-driven transcriptional programs but is also strongly modulated by baseline inflammatory burden and concomitant disease-modifying antirheumatic drug use, reducing sensitivity to modest immunologic shifts over short intervention windows [20,57].

Our results showed that probiotics significantly reduced the levels of hs-CRP in patients with MS, an inflammatory and autoimmune neurological disorder which leads to demyelination. Though not as much research has been published on the effect of probiotics in patients with MS as for patients with RA, the overall results are encouraging. In 2019, Morshedi et al. [58] reviewed studies of the effects of probiotics on MS and concluded that probiotics could improve immune and inflammatory parameters in patients with MS. In a similar review published in 2021, Blais et al. [59] included 6 human and 31 animal studies examining the effect of probiotics on MS. The authors concluded that the most promising probiotics were formulations containing *Lactobacillus paracasei*, *Bifidobacterium animalis*, *E. coli* Nissle 1917, and *Prevotella histicola*. Tsogka et al. [60] reviewed 13 studies published between 2011 and 2023, and found that a probiotic mixture showed promising results on MS-related fatigue, Expanded Disability Status Scale parameters, and inflammation. Overall, the authors concluded that probiotics promote a shift in inflammation towards an anti-inflammatory cytokine profile, as well as improving disability, mood, and quality of life.

As previously discussed, it is believed that probiotics improve the gut microbiota and thus influence immune function and systemic inflammation. A recent systematic review and meta-analysis suggested that probiotics may improve intestinal barrier integrity, and thus reduce the translocation of endotoxins and reduce systemic inflammation [61]. The idea of the “gut–brain” axis has recently been postulated to understand how altering the gut microbiota may improve neurological conditions such as MS [62,63]. A mechanistically plausible explanation for the greater hs-CRP improvement observed with probiotic supplementation in the MS subgroup is the mitigation of MS-associated intestinal barrier dysfunction and low-grade microbial translocation: elevated circulating endotoxin (lipopolysaccharide) has been reported in MS and is related to IL-6 production and heightened T helper 17-like responses [64]. By restoring microbial ecology and enhancing barrier integrity, probiotics may reduce endotoxin-driven Toll-like receptor signaling and downstream nuclear factor kappa B activation, thereby attenuating upstream cytokine cues—particularly IL-6—that drive hepatic acute-phase gene programs, including *C*-reactive protein transcription via signal transducer and activator of transcription 3-dependent pathways [57]. In parallel, probiotic-induced increases in immunoregulatory microbial metabolites (for example, short-chain fatty acids) may promote Treg differentiation and strengthen barrier and blood–brain barrier homeostasis, further dampening systemic inflammatory tone [65]. This pathway-level rationale is consistent with randomized trials and evidence syntheses in MS showing favorable shifts in inflammatory markers after probiotic supplementation, supporting hs-CRP as a responsive systemic readout when baseline gut-driven inflammation is present [66].

In addition, clinical evidence for isolated prebiotic oral supplementation in autoimmune diseases remains very limited. Although recent trials in rheumatoid arthritis and type 1 diabetes suggest potential benefits on *C*-reactive protein, *C*-peptide, gut permeability, and microbiota composition, these studies did not provide a complete evaluation of the inflammatory and oxidative stress markers analyzed in the present review, including interleukin-6, interleukin-10, interleukin-1 beta, tumor necrosis factor alpha, high-sensitivity *C*-reactive protein, malondialdehyde, and total antioxidant capacity [67,68,69]. Despite this clinical gap, prebiotics remain mechanistically plausible because fermentation-derived short-chain fatty acids can improve barrier integrity and regulate immune tolerance through effects on regulatory T cells, T helper 17 responses, and inflammatory signaling pathways [70,71,72]. This rationale is supported by preclinical autoimmune models in multiple sclerosis, arthritis, and lupus, but dedicated human randomized controlled trials of stand-alone prebiotics with prespecified biomarker endpoints are still needed [73,74,75,76].

Although the present review primarily synthesized biomarker outcomes, the available literature suggests that oral probiotic supplementation may also influence clinical symptoms in selected autoimmune diseases; however, these data were reported less consistently than laboratory endpoints in the included trials. In inflammatory arthritis, a systematic review found small but statistically significant improvements in quality of life and pain, while also emphasizing substantial heterogeneity in probiotic formulations, treatment duration, and study populations [22]. The strongest symptom-based evidence appears to come from rheumatoid arthritis, in which *Lactobacillus casei* 01 supplementation reduced tender and swollen joint counts, improved global health scores, lowered the Disease Activity Score in 28 joints (DAS28), and increased the proportion of patients achieving a moderate European League Against Rheumatism response [28]. However, this apparent benefit should be interpreted cautiously, because a later meta-analysis reported that, after the exclusion of high-risk-of-bias trials, the effect of probiotics on the DAS28 was no longer statistically significant [24]. In multiple sclerosis, a randomized clinical trial showed that Saccharomyces boulardii supplementation significantly reduced pain intensity and fatigue severity and improved selected domains of quality of life, suggesting that probiotic-related benefits may extend beyond biomarker modulation alone [29]. In plaque psoriasis, a randomized double-blind trial demonstrated improvement in the Psoriasis Area and Severity Index, Psoriasis Symptom Scale, Dermatology Life Quality Index, and depressive symptom scores after probiotic supplementation [35]. Nevertheless, more recent pooled evidence indicates that, although probiotics may improve the Psoriasis Area and Severity Index, improvement in the Dermatology Life Quality Index has not been shown consistently across trials [77]. By contrast, evidence in systemic lupus erythematosus remains preliminary and is derived mainly from synbiotic, rather than isolated probiotic, interventions; although improvement in the Systemic Lupus Erythematosus Disease Activity Index 2000 has been reported, the independent contribution of probiotics cannot be clearly separated [38]. Taken together, current evidence suggests that probiotics may improve patient-important outcomes in some autoimmune diseases, particularly rheumatoid arthritis, multiple sclerosis, and psoriasis, but the small sample sizes, short follow-up periods, and heterogeneity of symptom measures preclude firm conclusions regarding the magnitude, durability, and generalizability of clinical benefit.

### 4.1. Clinical Implications and Future Directions

The findings of this meta-analysis suggest that oral probiotic supplementation could serve as a complementary therapy in the management of autoimmune diseases, particularly for patients with RA and MS. Given the significant reduction in inflammatory markers, probiotics may help to modulate disease activity and improve long-term outcomes. Clinically, probiotics offer a non-invasive, relatively low-risk intervention supplemental to traditional immunosuppressive therapies. However, the variability in response across different autoimmune conditions suggests that probiotics may not be universally effective and should be considered as part of a tailored therapeutic approach. Future studies should aim to explore the long-term effects of probiotics on disease activity and remission rates in patients with autoimmune diseases. Further RCTs with larger sample sizes and longer follow-up periods are needed to confirm the effects of probiotics and identify the mechanistic pathways involved. Additionally, research into specific strains of probiotics and their differential effects on autoimmune diseases could provide more targeted therapeutic options. Lastly, exploring the role of probiotics in combination with dietary modifications and other non-pharmacological interventions may reveal synergistic effects.

### 4.2. Strengths and Limitations

This systematic review and meta-analysis has several strengths and limitations. Among its strengths is a comprehensive literature review ensuring that a wide range of relevant studies on probiotics and autoimmune diseases were included. The focus on RCTs minimizes bias, enhancing the reliability of the findings. Additionally, the analysis encompassed diverse patient populations, providing insights into the potential benefits of probiotics across various autoimmune conditions, particularly RA. However, this systematic review and meta-analysis also has limitations. Heterogeneity was observed across the included trials in terms of sample size, intervention characteristics (probiotic strains/formulations and dosage), and treatment duration, which may limit the generalizability of the pooled estimates. In addition, most studies primarily reported laboratory biomarkers (e.g., inflammatory cytokines and hs-CRP) and provided limited data on clinical symptoms or disease activity, thereby restricting inferences regarding any disease-modifying effect of probiotic-related supplementation. Further, no eligible trial evaluated isolated prebiotic formulations, and findings are therefore driven primarily by probiotic or synbiotic regimens. Although several primary biomarkers showed statistically significant changes, oxidative stress-related secondary outcomes (MDA and TAC) were not consistently improved, warranting further investigation. Finally, follow-up periods were generally short, precluding robust assessment of sustained or long-term effects.

## 5. Conclusions

In conclusion, this systematic review and meta-analysis provides compelling evidence that oral probiotic supplementation may be an effective adjunctive therapy for managing inflammation in autoimmune diseases, particularly RA. The significant reductions observed in key inflammatory markers highlight the potential benefits of probiotics in enhancing immune regulation and alleviating symptoms. However, given the heterogeneity among studies and some methodological limitations, further research with standardized protocols and longer follow-up periods is needed to fully establish the efficacy and safety of probiotics across different autoimmune conditions. Overall, probiotics represent a promising avenue for non-invasive therapeutic strategies in the management of autoimmune diseases.

## Data Availability

The original contributions presented in this study are included in the article/Supplementary Material. Further inquiries can be directed to the corresponding author.

## Supplementary Materials

The following supporting information can be downloaded at: https://www.mdpi.com/article/10.3390/nu18071107/s1, Figure S1: The quality assessment was using the Cochrane Collaboration tool, the risk of bias is presenting by Risk of bias graph (A) and Risk of bias summary (B).
