# Supplementary figures and images for: Effect of Probiotic and Synbiotic Oral Supplementation in Autoimmune Diseases: An Updated Systematic Review and Meta-Analysis of Randomized Controlled Trials
Source: Nutrients. 2026 Mar 30;18(7):1107. doi: 10.3390/nu18071107 (PMC13074674; doi:10.3390/nu18071107)

Supplementary Figure S1

A

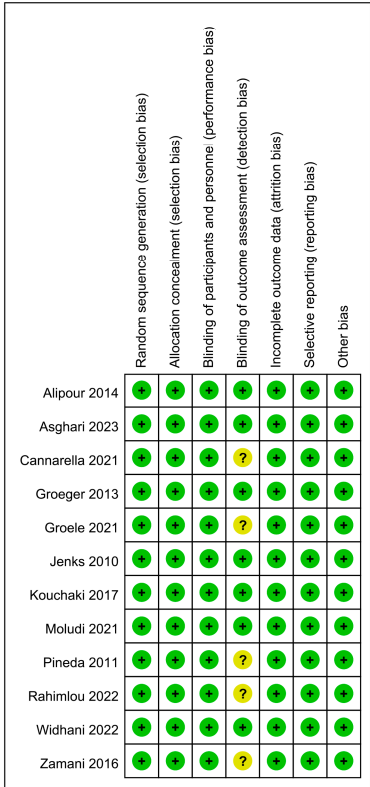

B

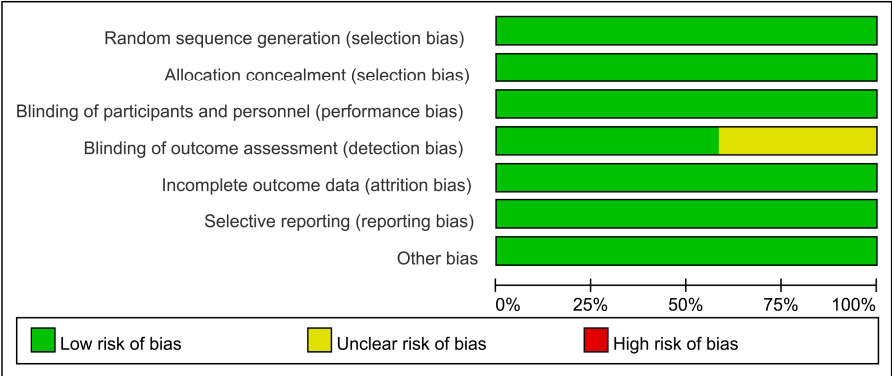

Supplement: Supplementary file 1 [file nutrients-18-01107-s001.zip › nutrients-4037719-supplementary (1).pdf]
